# Synergistic Surface Modification for High‐Efficiency Perovskite Nanocrystal Light‐Emitting Diodes: Divalent Metal Ion Doping and Halide‐Based Ligand Passivation

**DOI:** 10.1002/advs.202305383

**Published:** 2023-11-30

**Authors:** Woo Hyeon Jeong, Seongbeom Lee, Hochan Song, Xinyu Shen, Hyuk Choi, Yejung Choi, Jonghee Yang, Jung Won Yoon, Zhongkai Yu, Jihoon Kim, Gyeong Eun Seok, Jeongjae Lee, Hyun You Kim, Henry J. Snaith, Hyosung Choi, Sung Heum Park, Bo Ram Lee

**Affiliations:** ^1^ School of Advanced Materials Science and Engineering Sungkyunkwan University Suwon 16419 Republic of Korea; ^2^ Department of Chemistry Research Institute for Convergence of Basic Sciences and Research Institute for Natural Science Hanyang University Seoul 04763 Republic of Korea; ^3^ Department of Physics Pukyong National University Busan 48513 Republic of Korea; ^4^ CECS Research Institute Core Research Institute Busan 48513 Republic of Korea; ^5^ Clarendon Laboratory Department of Physics University of Oxford Oxford OX1 3PU UK; ^6^ Department of Materials Science and Engineering Chungnam National University Daehak‐ro, Yuseong‐gu Daejeon 34134 Republic of Korea; ^7^ Institute for Advanced Materials and Manufacturing Department of Materials Science and Engineering University of Tennessee Knoxville TN 37996 USA; ^8^ School of Earth and Environmental Sciences Seoul National University Seoul 08826 Republic of Korea

**Keywords:** ligand engineering, metal ion, perovskite light‐emitting diodes, perovskite nanocrystals, surface passivation

## Abstract

Surface defects of metal halide perovskite nanocrystals (PNCs) substantially compromise the optoelectronic performances of the materials and devices via undesired charge recombination. However, those defects, mainly the vacancies, are structurally entangled with each other in the PNC lattice, necessitating a delicately designed strategy for effective passivation. Here, a synergistic metal ion doping and surface ligand exchange strategy is proposed to passivate the surface defects of CsPbBr_3_ PNCs with various divalent metal (e.g., Cd^2+^, Zn^2+,^ and Hg^2+^) acetate salts and didodecyldimethylammonium (DDA^+^) via one‐step post‐treatment. The addition of metal acetate salts to PNCs is demonstrated to suppress the defect formation energy effectively via the ab initio calculations. The developed PNCs not only have near‐unity photoluminescence quantum yield and excellent stability but also show luminance of 1175 cd m^−2^, current efficiency of 65.48 cd A^−1^, external quantum efficiency of 20.79%, wavelength of 514 nm in optimized PNC light‐emitting diodes with Cd^2+^ passivator and DDA ligand. The “organic–inorganic” hybrid engineering approach is completely general and can be straightforwardly applied to any combination of quaternary ammonium ligands and source of metal, which will be useful in PNC‐based optoelectronic devices such as solar cells, photodetectors, and transistors.

## Graphical abstract

A synergistic metal ion doping‐surface ligand passivation strategy is developed to effectively passivate the surface defects of CsPbBr_3_ perovskite nanocrystals (PNCs) via didodecyldimethylammonium organic ligands and divalent metals dopant. This “organic–inorganic” hybrid engineering approach can be straightforwardly applied to any combination of quaternary ammonium ligands and source of metal, which will be useful in PNC‐based optoelectronic devices.

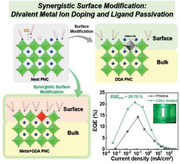

## 1 Introduction

Lead halide perovskite nanocrystal (PNC) light emitting diode (LED) technology has improved rapidly in recent years for next‐generation RGB full‐color display technology owning to their outstanding optoelectrical properties including tunable emission wavelength with varying halide components or size, high color purity with narrow emission spectra and high photoluminescence quantum yields (PLQYs).^[^ ^1^, ^2^, ^3^, ^4^, ^5^, ^6^, ^7^, ^8^ ^]^ Although bulk perovskites are expected to be defect‐tolerant, as‐synthesized PNCs are capped with long organic chain ligands such as oleate (OA^−^) and oleylammonium (OAm^+^) for monodisperse and uniformly shaped PNC synthesis; the weak binding ability of native OA^−^/OAm^+^ causes dynamic binding of these ligands, causing poor colloidal stability and high surface defect densities in terms of halide vacancies and undercoordinated lead atoms.^[^ ^9^ ^‐^ ^15^ ^]^

Over the past few years, substantial effort has been put on to tackle this problem by removing either of the two surface defects caused by this phenomenon, namely the A‐site defects (mostly via ligand engineering involving quaternary ammonium ions) and B‐site defects (via metal ions and chelates).^[^ ^16^, ^17^, ^18^, ^19^ ^]^ Both types of defects are known to induce a number of generally undesirable features in perovskite‐based optoelectronics such as halide migration, poor environmental stability, and short carrier lifetime. In terms of PNC‐based LEDs specifically, these surface defects most often manifest themselves as nonradiative recombination of PNCs; together with the severely compromised carrier injection, they play a determining role in the performance and operational stability of these devices.^[^ ^20^, ^21^ ^]^

Various strategies have thus been utilized over the years to address this issue of surface defects in PNCs. An approach well‐known in the field is ligand engineering of surface‐attached ligands to mitigate the issue of dynamic binding: organic ligands such as ammonium halides, amino acids, benzoyl halides, chelating ligands, and many others have been explored to this end.^[^ ^4^, ^22^, ^23^, ^24^ ^]^ Of these, didodecyldimethylammonium halide (DDAX, X = F, Cl, Br, I) ligands have been successfully demonstrated to increase the efficiency of LEDs by passivating the PNC surface.^[^ ^25^, ^26^, ^27^ ^]^

On the other hand, a B‐site engineering strategy with divalent metal dopant cations has successfully been used to control B‐site vacancies stabilize surface, and reduce the defect density of PNCs, where the divalent metal ions are known to strengthen the bonding with surface ligands and prevent the desorption of organic ligands.^[^ ^28^, ^29^ ^]^ In particular, the incorporation of small B‐site cations such as Cd^2+^ has been shown as a strategy to better accommodate the relatively large quaternary ammonium ligands by partially distorting the near‐surface [MX_6_]^[^ ^4^ ^]−^ octahedra to render large‐sized A‐site vacancies, providing us with an interesting example of how the A‐ and B‐site species can effectively influence each other during passivation.^[^ ^25^ ^]^ In fact, this interaction can also work in a reverse manner such that the binding of DDA onto perovskite surfaces can cause a similar distortion in the perovskite lattices, thus creating B‐site cation vacancies in the process. Undoubtedly, this behavior will again lead to undesirable nonradiative recombinations which, in part, curtails the efficacy of DDA treatments. Clearly, a cooperative passivating strategy to manage both the A‐ and B‐site defects, preferably through a simplified synthetic protocol, would be an elegant solution to such a problem.

Starting from this motivation, our current investigation attempts to exploit the synergistic surface interaction between metal dopants (Zn, Cd, Hg, and Pb) and organic ammonium ligands (DDA) to provide a solution to this issue. We introduce a hybrid surface ligand exchange process by use of metal acetates and DDA ligands to allow a denser binding of organic ligands as well as lower halide defect formation energy, all of which can inhibit the nonradiative recombination in Cs‐based green emissive PNCs. The resulting PNCs with cadmium acetate and DDA‐organic ligands showed a 96.02% PLQY at 514 nm, compared to 41.77% of as‐synthesized PNCs and 67.18% of DDA‐PNCs. When applied in LED devices, the optimized device with synergetically passivated PNCs displayed a current efficiency of 65.48 cd A^−1^ and an efficient external quantum efficiency (EQE) of 20.79% in the green region. Our present approach of “organic–inorganic” surface engineering can thus be an effective strategy for suppressing surface defects for PNCs and can be extended to other fields of perovskite‐based electric devices where controlling the defects is deemed crucial.

## 2 Results and Discussion

### 2.1 Ab Initio Screening of Metal Ion Dopants

**Figure 1:**
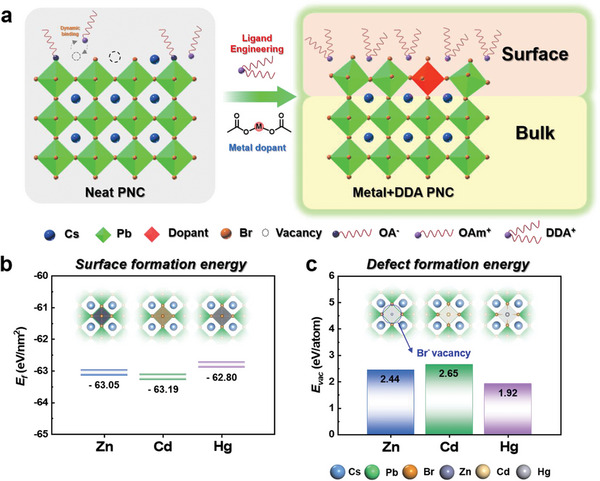
a) Schematic illustration of the hybrid inorganic and organic ligand engineering process. b) DFT‐estimated Surface formation energy and c) DFT‐calculated defect formation energy of each metal onto the CsBr‐terminated CsPbBr_3_ surfaces.

The “organic–inorganic” passivated CsPbBr_3_ PNCs were synthesized by a solution‐based ligand exchange method following the standard hot‐injection method, similar to our previous reports.^[^ ^11^, ^30^ ^]^ **Figure** **1**a shows a schematic illustration of our approach. A key difference in this study was to add the metal ions simultaneously to the exchange solution to induce their incorporation into PNC surfaces (see the Experimental Section for details). Metal acetate precursors with varying sizes of transition metal ions, all belonging to group 12 with smaller sizes than Pb^2+^ (119 pm), were used as metal dopants: Zn^2+^ (74 pm), Cd^2+^ (95 pm) and Hg^2+^ (102 pm). As outlined in the Introduction, the sizes of metal atoms are expected to play a vital role in determining the structural outcomes of doping, alongside the binding of organic ligands. Ab initio calculations have allowed us to gain theoretical insights into such atomic‐scale scenarios taking place at the PNC surface.^[^ ^4^, ^11^, ^15^, ^30^ ^]^ In light of this, to screen the effect of metal additives on the surface stability of PNCs, we calculated the surface formation energy (*E_f_*) and the defect formation energy (*E_vac_*) of a Br ion with three different divalent cations (Figure 1b,c). The negative *E_f_* values in Figure 1b indicate that substituting Pb for Zn, Cd, and Hg is thermodynamically feasible. Interestingly, the Cd‐treated CsBr‐terminated surface exhibits the lowest *E_f_* of −63.19 eV nm^−2^ and the highest *E_vac_* of 2.65 eV atom^−1^ (refer to Supplementary information for detailed methods). The calculated *E_vac_* from DFT suggests that the Cd‐passivated PNC is relatively more resistant to charge recombination than the Zn‐ or Hg‐passivated counterparts (Figure 1c). In addition, the substitutional energy (*E_sub_*), which scales the thermodynamic driving force of doping, shows that the Cd^2+^ ion relatively well stabilizes the structural framework of the PNC structure more than Zn^2+^ or Hg^2+^ ions (Figure S2, Supporting Information). Furthermore, the Cd‐passivated PbBr_2_‐terminated surface of PNC also shows the highest defect resistance and structural stability than the Zn‐ or Hg‐passivated surfaces (Figure S3, Supporting Information). Thus our DFT calculation results predict that cadmium passivation would provide the best passivation of the Pb‐vacancy site and improve the structural stability of PNCs, a prediction which we now attempt to confirm experimentally.

### 2.2 Structural and Optical Properties of Organic–Inorganic Engineered PNCs: the Inorganic Metals

**Figure 2:**
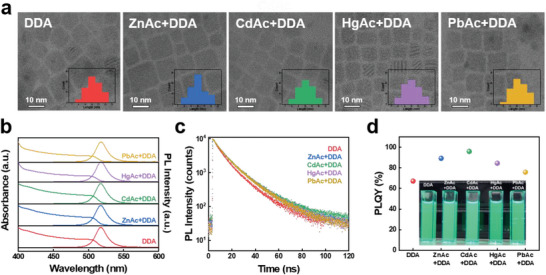
a) High magnification TEM image, b) Absorption and PL spectra, c) Time‐resolved PL decay curves, d) PLQY of PNCs dispersions.

Transmission electron microscope (TEM) images and optical measurements of the synthesized PNCs are shown in **Figure** **2**. Size analysis of TEM images revealed that the PNCs retained the original size (≈10 nm) and cubic shape after the organic–inorganic ligand exchange process when compared to neat PNCs (still capped with OA^−^ and OAm^+^ ligand; see Figures S4 and S5, Supporting Information). The TEM image of each PNCs exhibit the uniform cubic shape with even size edge length: 10.30 ± 0.99 (DDA), 10.07 ± 0.99 (ZnAc+DDA), 10.02 ± 1.10 (CdAc+DDA), 9.98 ± 1.08 (HgAc+DDA), and 10.29 ± 1.11 (PbAc+DDA) nm, respectively. Low magnification TEM images of the metal treated with DDA ligand PNCs are shown in Figure S6a (Supporting Information), and the corresponding EDS mapping images of Cs, Pb, Zn, Cd, Hg, and Br atoms overlap well with the TEM images indicating the presence of each metal in the PNC (Figure S6b, Supporting Information).

Subsequently, powder X‐ray diffraction (PXRD) was performed on the PNCs to detect any structural changes upon the addition of metal ions. The results from the PXRD pattern (Figure S7, Supporting Information) showed that there was no shift in the diffraction angle, indicating that the metal ions remain on the surface of the PNCs instead of being integrated into the bulk perovskite lattice.^[^ ^11^, ^17^ ^]^ Furthermore, X‐ray photoelectron spectroscopy (XPS) measurement of each PNCs was performed to explore their chemical states (Figure S8, Supporting Information). In the XPS spectra, 284.8 eV peak represents of C─C bond of perovskite and surface contamination (Figure S8a, Supporting Information).^[^ ^31^ ^]^ Furthermore, the N 1s peak appears at 402.2 eV due to PNCs containing DDA (Figure S8b, Supporting Information).^[^ ^32^ ^]^ All PNCs showed clear signals of Cs 3d, Pb 4f, and Br 3d, respectively (Figure S8c–e, Supporting Information). However, no noticeable differences were observed in the XPS spectra of each PNC. As confirmed with XPS, the actual concentration of each metal ion to CsPbBr_3_ NCs indicates the doping level of metal is relatively low (Table S1, Supporting Information). Despite the small amount of metal passivating agent, we expect that this small concentration is sufficient to fill the B‐site vacancies on the PNC surface and passivate the relevant defects.^[^ ^11^ ^]^

All green emissive CsPbBr_3_ PNCs (DDA PNC and metal acetate‐treated PNCs with DDA ligand) exhibit identical UV–vis absorption and photoluminescence (PL) spectra centered at 514 nm (Figure 2b). These results indirectly support the surface passivating nature of metal ions, rather than being incorporated in the bulk as dopants, which would subsequently change the bulk electronic structure and absorption curves.^[^ ^17^, ^33^, ^34^ ^]^ To study the effectiveness of passivating surface defects on PNCs through the additional metal acetate treatment, we conducted time‐resolved photoluminescence decay (TRPL) and PLQY measurements (Figure 2c,d). PL decay curves were fitted by a multi‐exponential function and the detailed data is summarized in Table S2 (Supporting Information). The PL average lifetime (*τ*_avg_) of PNC solutions was increased from 9.04 (DDA) to 10.68 (ZnAc+DDA), 10.80 (CdAc+DDA), 10.63 (HgAc+DDA), 10.23 ns (PbAc+DDA) respectively. The PLQY of the DDA and each metal‐treated NCs solution were measured as 67.18% (DDA), 89.28% (ZnAc+DDA), 96.02% (CdAc+DDA), 84.49% (HgAc+DDA), and 75.80% (PbAc+DDA). Strong agreement between the TRPL and PLQY data signifies stronger radiative recombination and reduced surface trap densities for the metal‐passivated PNCs, Cd‐passivated PNCs being the best available candidates which we will focus on from now on.

### 2.3 Organic–Inorganic Interaction and Environmental Stability: the Organic Ligands

As outlined in the Introduction, surface inorganic metal ions can play a decisive role in determining the surface binding of organic ligands (DDA in the current case), which directly influences the surface density of organic ligands on the PNCs. This is crucial because these surface ligands are key to achieving high device efficiencies through defect suppression, alongside bestowing long‐term stability by blocking the penetration of environmental moisture, the main culprit for surface‐initiated degradation of PNCs.

While the inorganic components within PNCs have been analyzed above using XPS, the organic ligands are best observed with Fourier‐transform infrared (FT‐IR) spectroscopy to look at characteristic functional group signals (Figure S9, Supporting Information). FT‐IR peaks located at 2926 and 2853 cm^−1^ are generally ascribed to the alkyl C─H stretches, present in all OA^−^, OAm^+^ and DDA ligands. The 1537 cm^−1^ peak (carboxyl C═O stretches) from OA^−^ and 1633 cm^−1^ peak (ammonium ─NH bending) from OAm^+^ are observed in the FT‐IR spectra of neat PNCs but not in the DDA and Metal+DDA PNCs.^[^ ^9^, ^26^ ^]^ These result as a whole indicates a successful ligand exchange from the native ligand (OA^−^/OAm^+^) to the DDA ligand. Of note, the absence of carboxylate peaks in the exchange PNCs also indicates the non‐binding nature of acetates contained in the metal precursor; these ions are presumably washed away during the post‐synthetic purification step using methyl acetate.

**Figure 3:**
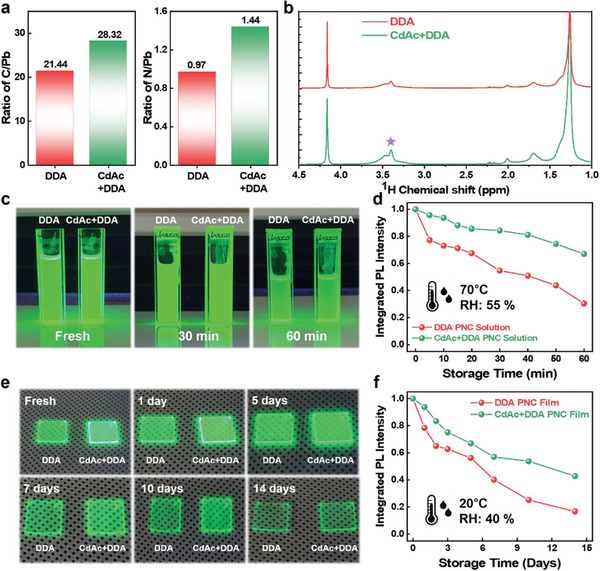
Estimated a) C to Pb and N to Pb elemental ratio of the PNC films based on XPS analysis. b) ^1^H NMR spectra of the DDA and CdAc+DDA PNCs dispersed in CDCl_3_. c) Photographic images of PNC solutions under 365 nm UV lamp illumination. d) Traces of integrated PL intensity of PNC solutions as a function of 70 °C storage time. e) Photographic images of PNC films under 365 nm UV lamp illumination. f) Traces of integrated PL intensity of PNC films as a function of ambient storage time.

However, quantitative estimation of the ligand density on surfaces using FT‐IR data is not straightforward due to issues with normalizing the observed FT‐IR intensities. For this purpose, we employ XPS data (at the cost of losing functional group resolution) since XPS is element‐specific and is (semi‐)quantitative between different elements. Here we use nitrogen (N) and carbon (C) 1s signals as proxies for quantities of DDA ligands since DDA is known to be the only existing organic ligand from FT‐IR analysis above. Therefore, the collective peak ratio of Pb 4f to N 1s and C 1s XPS signals in a PNC can be used to quantify the surface ligand density.^[^ ^35^, ^36^ ^]^ **Figure** **3**a presents the ratio of N and C to Pb obtained from XPS analysis. Compared to the C/Pb and N/Pb ratio obtained with DDA PNC, the ligand content of CdAc+DDA PNCs is approximately 1.32 times (C/Pb) and 1.49 times (N/Pb) higher, indicating clear evidence of denser ligand binding on the surface of the nanocrystal.

Another method of quantifying the ligand density is NMR spectroscopy, in which the peak intensity is directly proportional to the number of spins present in the sample. In this case, quantitative ^1^H solution‐state NMR of dissolved PNCs can also provide insight into the relative concentration of ligands in each sample. Two spectra were acquired under identical conditions by dispersing 6 mg of PNCs and 0.5 mg of ferrocene in 1 mL CDCl_3_ solutions. Integration of the peak areas corresponding to the alkyl environments attached to nitrogen (N─CH_2_─ and N─CH_3_, 4.3 to 3.0 ppm region),^[^ ^25^, ^37^ ^]^ together with the sizes of PNCs measured from TEM images, yields a quantitative estimation of DDA densities as 1.49 and 2.31 molecules nm^−2^ for the DDA‐only and CdAc+DDA samples, respectively. This ≈1.6‐fold increase in DDA density is in good agreement with the XPS signal ratio (1.3 and 1.4 times for C/Pb and N/Pb). Thus the two data confirm our hypothesis of significantly denser DDA ligands on CdAc+DDA PNCs and are shown to be the basis for the improved optical properties as well as colloidal and environmental stability of PNC films which we now discuss.

To investigate this phenomenon, aliquots of each nanocrystal solution were heated to 70 °C, and the resulting PL intensity was monitored (Figure 3c,d). At this elevated temperature, facile detachment of ligands from the surface of the nanocrystals would lead to a loss of colloidal stability and a decrease in PL intensity. However, our results show that the CdAc+DDA nanocrystals have better colloidal and PL stability than the ones treated solely with DDA due to the reduced detachment of ligands from the surface, clearly showing the effectiveness of organic–inorganic hybrid passivating strategy on colloidal stability.

Finally, we investigated the stability of PNC films with DDA PNC and CdAc+DDA PNC. All PNC films are deposited on the glass substrate and stored at ambient conditions (40% relative humidity, room temperature 20 °C, and the initial PLQY values of each PNC film shown in Figure S10, Supporting Information). Figure 3f shows the time evolution of integrated PL intensity results of each PNC film and photos of PL under UV lamp illumination (Figure 3e). DDA PNC film showed rapid degradation within 7 days, due to surface vacancies, but CdAc+DDA PNC films showed stable emission even after 14 days (≈50% of the initial intensity). In turn, this corroborates our hypothesis that the metal cation decoration enables stronger ligand passivation of the PNC surface by DDA.

### 2.4 Defect Density and PeLED Device Performance of Organic–Inorganic Engineered PNCs

To explore how the synergistic organic–inorganic surface passivation can control the defect densities of the PNC films, *J–V* characteristics of the corresponding hole‐only devices (ITO / HTLs / PNCs / MoO_3_ / Ag; Figure S11a, Supporting Information) were evaluated. Trap‐filled‐limited voltage (V_TFL_) was obtained from the *J–V* curve of the hole‐only device (Figure S11b, Supporting Information), where a lower *V*_TFL_ value means a lower trap density in the PNCs.^[^ ^30^ ^]^ V_TFL_ was reduced from 0.42 V (DDA) to 0.26 V (CdAc+DDA). (Details in Figure S11, Supporting Information) The trap state density of PNCs can be determined by the following equation.^[^ ^38^ ^]^

(1) $$N_{t}=\frac{2\varepsilon \varepsilon_{0}V_{\mathrm{TFL}}}{eL^{2}}$$

*ε* and *ε_0_* are the dielectric constant of CsPbBr_3_ (4.96)^[^ ^1^ ^]^ and vacuum permittivity (8.854 × 10^−12^ F m^−1^)^[^ ^36^ ^]^ *e* is the elementary charge (1.6 × 10^−19^ C)^[^ ^39^, ^40^ ^]^ and *L* is the thickness of the PNC film (≈40 nm). Calculated defect densities (*N*_t_) of DDA and CdAc+DDA PNCs are 1.44 × 10^17^ cm^−3^ and 8.92 × 10^16^ cm^−3^, showing the effectiveness of our synergistic organic–inorganic surface passivation strategy.

**Figure 4:**
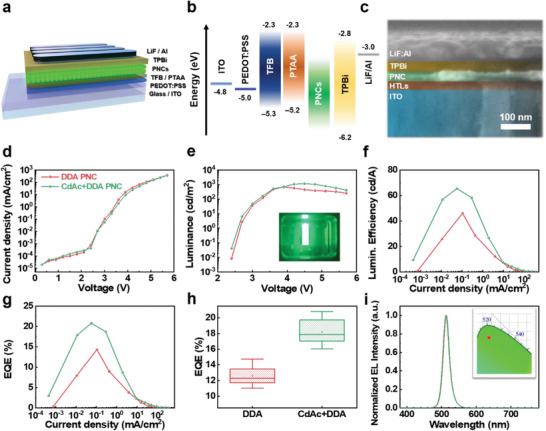
a) Device architecture, b) Energy level diagram, c) Cross‐sectional SEM image of a PeLED device. d) Current density–voltage (*J–V*) curves. e) Luminance–voltage (*L–V*). f) Current density–Luminance efficiency (*J–LE*). g) Current density–EQE (*J–EQE)*. h) *EQE*_max_ histogram of each based on the 15 individual devices. i) EL spectra at *EQE*_max_ of PeLED and CIE coordinate of the EL spectra (inset).

To confirm the performance of PNCs on the optoelectronic device, we fabricated PeLEDs with DDA PNC and various metal‐treated PNCs. The LED device architecture and PeLED's energy level diagram in this work are shown in **Figure** **4**a,b. The energy level of each layer was according to previous reports^[^ ^3^, ^30^ ^]^ and ultraviolet photoelectron spectroscopy (UPS) results (Figure S12, Supporting Information). Figure 4c shows a cross‐sectional scanning electron microscope (SEM) image of PeLED about the following structure; ITO (150 nm) / HTLs (PEDOT: PSS/TFB/PTAA 35 nm) / Perovskite NCs (40 nm) / TPBi (40 nm) / LiF (1 nm) / Al (100 nm).

Figure 4d–i shows the device performances; d) Current density versus voltage, e) Luminance versus voltage, f) Current density versus luminance efficiency, g) Current density versus EQE, h) EQE value histogram of each based on the 15 individual devices, respectively.

Comparing the LED performance of DDA PNC device and champion device (CdAc+DDA PNC), the effect of hybrid organic–inorganic engineering is obvious (Figure 4h): DDA PeLED shows maximum luminance and EQE_max_ of 684 cd m^−2^ and 14.32%, while the champion PeLED with CdAc+DDA shows 1175 cd m^−2^ and 20.79% with reliable reproducibility (**Table** **1**). The details of each PeLED performance parameter are summarized in Figure S13 and Table S3 (Supporting Information). Each PeLED device shows an EL spectrum at 514 nm with 20 nm full width at half‐maximum (FWHM) which is the same as the PL spectrum (Figures 2b and Figure 4i). In addition, the EL spectra of the champion device exhibit stable EL spectrum at 514 nm at each with voltage from 2.7 to 4.5 V (Figure S14, Supporting Information).

**Table 1:** Summarized device performances with DDA and CdAc+DDA PNC champion devices.

Sample configuration	L_max_ [cd m^−2^] @ bias	LE_max_ [cd A^−1^] @ bias	EQE_max_ [%] @ bias	Turn‐on Voltage[V] @ [1cd m^−2^]	Wavelength [nm]
DDA PNC	530@3.6	47.68@3.0	14.72@3.0	2.7	514
CdAc+DDA PNC	1175@4.5	65.48@3.0	20.79@3.0	2.7	514

As confirmed by the PL stability of each PNCs under ambient conditions (Figure 3e,f), further investigation of the DDA PNC and CdAc+DDA PNC device stability was performed by lifetime measurement (T_50_) and joule heat control according to PNC's stability (Figure S15a–f, Supporting Information). Figure S15a,d (Supporting Information) shows the DDA PNC and CdAc+DDA PNC device's operational stability under constant current densities of 0.03, ≈0.60, ≈1.7 mA^−2^, respectively. CdAc+DDA PeLEDs have similar current densities with DDA LEDs but higher initial luminance values. The decay time to 50% (T_50_) from initial luminance in the CdAc device is 1.1, 2.1, 8.7 min showing around 1.3‐fold longer than the DDA PNC device (6.7 min, Figure S15a,d, Supporting Information). In addition, both devices show stable green emission of similar spectra at each point (*T*_100_, *T*_75_, *T*_50_, Figure S15b,e, Supporting Information). The temperature of both devices was measured with an IR thermal imaging camera during the device's operation (Figure S15c,f, Supporting Information). The DDA PNC and CdAc+DDA PNC device's operation temperature at 33 mA cm^−2^ were measured as 26.53 and 24.34 °C (at 4.5 V; maximum luminance voltage).

Therefore, these results can explain the filling of the surface defects on the PNC's surface and the reduction of lattice strain through metal treatment.^[^ ^41^, ^42^, ^43^ ^]^ Therefore, the thermal‐induced joule heating of PeLEDs can be also suppressed by the surface inorganic treatment.

## 3 Conclusion

In this work, we introduced an organic–inorganic hybrid passivating approach to reduce the surface defects in CsPbBr_3_ PNCs. By exploiting the synergistic interaction between the organic DDA ligand and inorganic metal species, we demonstrate that metal surface passivation can simultaneously provide an increased surface coverage of DDA ligands with more complete passivation of the surface. Such a dual passivation approach results in significantly enhanced environmental and colloidal stabilities of PNCs as well as near unity PLQY, which translates to highly efficient PeLED performances. Since surface defect passivation is a crucial issue that determines the properties of many perovskite‐based devices, we expect this approach to shed new insight on using multiple passivating agents, an increasingly common strategy to yield better defect suppression, through exploiting the interaction among the passivating species.

## 4 Experimental Section

### Materials

Cesium carbonate (99.9%), Cadmium acetate dihydrate (reagent grade 98%), Zinc acetate dihydrate (99.999% trace metals basis), Mercury acetate (ACS reagent ≥98.0%) 1‐octadecene (ODE; 90% tech.), Oleylamine (OAm; 70% tech.), Oleic acid (OA; 90% tech.), Methyl acetate (MeOAc; anhydrous 99.5%), Octane (anhydrous ≥99%), Toluene (anhydrous 99.8%), Hexane (anhydrous 95%), Chlorobenzene (anhydrous 99.8%), Lead bromide (PbBr_2_; 99.999% trace metal basis), poly[(9,9‐dioctylfluorenyl‐2,7‐diyl)‐co‐(4,4′‐(N‐(4‐sec‐butyl phenyl)diphenylamine)] (TFB) and Didodecyldimethylammonium bromide (DDAB; 98%) and sodium fluoride (NaF; BioXtra, ≥99%) were purchased from Sigma Aldrich. Poly[bis(4‐phenyl)(2,4,6‐trimethylphenyl)amine (PTAA; M_w_: 30 000) was purchased from Ossila. Indium tin oxide‐based transparent conductive electrode (≈4.5 Ω sq^−1^) purchased from AMG. MoO_3_ (99.9995%) was purchased from Alfa Aesar. Aqueous solution of PEDOT: PSS (Clevios AI 4083) purchased from Heraeus. 2,2′,2″‐(1,3,5‐benzinetriyl)tris(1‐phenyl‐1‐H‐benzimidazole) (TPBi; 99.9%) was purchased from OSM. LiF (99.9%), Ag (99.99%), and Al (99.9%) were purchased from iTASCO.

### Synthesis of CsPbBr_3_ PNCs

For the cesium‐oleate solution, 407 mg of cesium carbonate, 1.25 mL of oleic acid (OA), and 20 mL of 1‐octadecene (ODE) were added to a three‐neck round‐bottom flask and stirred under vacuum for 60 min at 120 °C for degassing to remove impurities. Then the temperature was risen to 150 °C for 30 min under N_2_ to completely make Cs‐oleate solution.

For CsPbBr_3_ NCs synthesis, 398 mg of PbBr_2_, 2.5 mL of OAm, 2.5 mL of OA, and 25 mL of ODE were stirred in a round‐bottom flask and degassed under vacuum at 120 °C for 60 min. The flask was then filled with N_2_. The temperature of the flask was risen to 150 °C for 30 min under N_2_ until the PbBr_2_ completely dissolved. Subsequently, the temperature of the PbBr_2_ solution was then increased to 180 °C. When the temperature of the PbBr_2_ solution reached 180 °C, 2 mL of Cs‐oleate was swiftly injected into the PbBr_2_ solution. After 10 s, the mixture solution was put into the ice bath to stop the reaction. When the temperature of the mixture solution was cooled down to 60 °C, then the light green colored CsPbBr_3_ nanocrystal crude solution was prepared.

### Purification

The crude solution was divided into two conical tubes of 50 mL volume ≈15 mL each, and precipitated by addition of 35 mL of MeOAc and then centrifuged at 8000 rpm for 3 min. After that, the supernatant was discarded, and precipitates of NCs from the centrifuge were redispersed in 5 mL of hexane. After that, 7.5 mL of MeOAc was added into the PNC solution and centrifuged again at 8000 rpm for 3 min. The final precipitates of NCs were redispersed in 5 mL of hexane and centrifuged again at 8000 rpm for 3 min. Final PNC solution filtered with PTFE filter (0.2 µm Whatman) to get a final solution.

### Preparation of Ligand Solution

In this method previously reported studies were followed.^[^ ^7^ ^]^ DDAB (231 mg, 0.5 mmol) and NaF (21 mg, 0.5 mmol) were dissolved in toluene (5 mL) and DI water (5 mL). The prepared solution was then reacted under sonication for 30 min. Then, the thick white mixture solution was centrifuged under 8000 rpm for 10 min, and finally, ligand solution in toluene was obtained.

### Preparing DDA and Metal Acetate‐Treated PNCs

In this procedure, 0.2 mL of ligand solution was added to the as‐synthesized PNC solution (8 mg mL^−1^ in Toluene) with stirring for 30 min at room temperature. For the metal acetate‐treated PNCs, 0.012 mmol of each metal acetate was added into PNC solution (8 mg mL^−1^ in Toluene) and add 0.2 mL of ligand solution and stirred for 30 min at room temperature. After the reaction, the mixture was centrifuged with MeOAc (reaction mixture: MeOAc is 1:3) at 9000 rpm for 3 min. The precipitates were redispersed in 1 mL of hexane for centrifugation again and then the supernatant was collected as the PNC solution.

### LED Device Fabrication

For PeLED devices, glass/ITO substrate was washed by ultrasonic treatment in deionized water, acetone, and 2‐propanol for 10 min each. After drying in an oven, the ITO substrates were treated with O_2_ plasma for 15 min. The aqueous PEDOT: PSS solution was spin‐coated on ITO at 4,500 rpm for 40 s and annealed at 140 °C on the hotplate for 10 min. After that, TFB (12 mg ml^−1^ in Xylene) and PTAA (14 mg mL^−1^ in Chlorobenzene) were spin‐coated at 4000 rpm for 40 s as following hole transport layers and annealed at 100 °C for 20 min in the nitrogen atmosphere individually. After annealed, PNC solution (dispersed in octane 5 mg mL^−1^) was spin‐coated at 2000 rpm for 40 s. Finally, TPBi (40 nm), LiF (1 nm), and Al (100 nm) were sequentially deposited at ≈10^−6^ Torr by the thermal evaporation method. The area of the Al electrode defines a 0.135 cm^2^ emission area of the device.

### Density Functional Theory

The performed spin‐polarized DFT calculations using a plane‐wave basis with the VASP code.^[^ ^44^ ^]^ The PBE (Perdew–Burke–Ernzerhof)^[^ ^45^ ^]^ generalized‐gradient‐approximation (GGA) level exchange‐correlation functional was applied for all calculations. The DFT‐D3 van der Waals correction method^[^ ^46^ ^]^ with the Becke–Johnson damping models was applied to improve the reliability of the results. The projector augmented wave (PAW) method^[^ ^47^ ^]^ described the interaction between the ionic core and the valence electrons. Valance electron wave functions were expanded on a plane‐wave basis up to an energy cutoff of 400 eV. The Brillouin zone was sampled at the Γ‐point for all calculations. The convergence criteria for the electronic structure and the atomic geometries were 10^−4^ eV and 0.05 eV Å^−1^, respectively. A Gaussian smearing function with a finite temperature width of 0.05 eV was used to improve the convergence of states near the Fermi level.

The optimized cell parameters of a cubic CsPbBr_3_‐ based PNC were a = b = c = 5.91 Å. Two types of structural models were constructed with different surface terminations, PbBr_2_‐ or CsBr‐terminated (3 × 3 × 6) slabs. Vacuum layers of 25 Å were applied on both sides of the slab to avoid the interaction between periodic cells. In addition, the atoms at two middle layers were fixed during the calculations.

The surface formation energy (*E_f_*), which represents the identical formation energy of a PNC model from atomic elements, was calculated as follows:

(2) $$E_{f}=\frac{E_{s}-\sum n_{i}\mu_{i}}{2A}$$

where *E_s_* represents the energy of the DFT‐constructed slab, *n*_i_ is the number of Cs, Cd, Hg, Zn, and Br atoms in a slab, and ε_i_ is the DFT‐calculated atomic energy of each element. The atomic energy of metallic elements was referenced to the corresponding bulk unit cell. The atomic energy of Br was referenced to the gas phase energy of Br_2_.

To estimate the structural stability of metal‐doped PNCs, the formulated the substitutional energy (*E_sub_*) and defect formation energy (*E_vac_*) as follows:

(3) $$E_{sub}=\frac{E_{doped}+nE_{Pb}-E_{pristine}-nE_{metal}}{n}$$

(4) $$E_{vac}=\frac{E_{de}-E_{pristine}+2E_{Br}}{2}$$

where the *E*_pristine_ is the system energy of the CsPbBr_3_ (100) surface, *E*_doped_ and *E*_vac_ represent the system energies with metal dopants and Br vacancy. *n* is the number of substitutional dopants, *E*_Pb_ and *E*_metal(Zn, Cd, Hg)_ are the energies of metal atoms referring to the bulk phase of each metal, and *E*_Br_ is the energy of the Br atom referring to the Br_2_ gas phase. The Br vacancy was introduced on both surface sides together to minimize the artificial dipole moment.

### Characterizations

SEM was measured with Verios G4 UC (FEI) and UPS spectra were collected using a photoelectron spectrometer (Thermo FisherScientific Theta Probe) with a He I (21.22 eV) ultraviolet source in Hanyang LNC 3.0 Analytical Equipment Center (Seoul). PLQY measurement was conducted with a Quantaurus‐QY Absolute PL quantum yield spectrometer (HAMAMATSU) equipped with an integrating hemisphere, and samples were excited at the wavelength of 365 nm. Steady‐state PL measurements were carried out using a pulsed xenon lamp. And time‐resolved PL decay measurements were carried out using a He–Cd laser operating at a wavelength of 375 nm. For PeLEDs, *J–V–L* characteristics and device performances were measured using a Konica Minolta spectroradiometer (CS‐2000) with a Keithley 2450 sourcemeter. XRD patterns were measured using an X'Pert‐MPD diffractometer (Philips, Netherlands) employing CuK*α* radiation. UV–vis absorption spectra were measured by a V‐770 spectrophotometer (JASCO). TEM samples were prepared by diluted QD solution in hexane dropped on a carbon grid. TEM was measured with the JEM‐2100F model (JEOL). The Fourier transform infrared (FT‐IR) was recorded on PerkinElmer Spectrum Two FT‐IR Spectrometer.

## Conflict of Interest

The authors declare no conflict of interest.

## Supporting information

Supporting Information

## Data Availability

The data that support the findings of this study are available in the supplementary material of this article.
