# Supplementary material for: Synergistic Surface Modification for High‐Efficiency Perovskite Nanocrystal Light‐Emitting Diodes: Divalent Metal Ion Doping and Halide‐Based Ligand Passivation
Source: Adv Sci (Weinh). 2023 Nov 30;11(4):2305383. doi: 10.1002/advs.202305383 (PMC10811502; doi:10.1002/advs.202305383)
Supplement: Supplementary file 1 — Supporting Information [file ADVS-11-2305383-s001.pdf]

ADVANCED SCIENCE
Open Access

Supporting Information

for *Adv. Sci.*, DOI 10.1002/advs.202305383

Synergistic Surface Modification for High-Efficiency Perovskite Nanocrystal Light-Emitting Diodes: Divalent Metal Ion Doping and Halide-Based Ligand Passivation

Woo Hyeon Jeong, Seongbeom Lee, Hochan Song, Xinyu Shen, Hyuk Choi, Yejung Choi, Jonghee Yang, Jung Won Yoon, Zhongkai Yu, Jihoon Kim, Gyeong Eun Seok, Jeongjae Lee, Hyun You Kim, Henry J. Snaith, Hyosung Choi, Sung Heum Park* and Bo Ram Lee**

Supporting Information

Synergistic Surface Passivation Effect for High Efficiency Perovskite Nanocrystal Light-Emitting Diodes: Divalent Metal Ion and Halide-based Ligand Passivation

Woo Hyeon Jeong, Seongbeom Lee, Hochan Song, Xinyu Shen, Hyuk Choi, Yejung Choi, Jonghee Yang, Jung Won Yoon, Zhongkai Yu, Jihoon Kim, Gyeong Eun Seok, Jeongjae Lee, Hyun You Kim, Henry J. Snaith, Hyosung Choi, Sung Heum Park* and Bo Ram Lee**

Dr. W. H. Jeong, Dr. X. Shen, Dr. Z. Yu, J. Kim, G. E. Seok, Prof. B. R. Lee
School of Advanced Materials Science and Engineering, Sungkyunkwan University, Suwon 16419, Republic of Korea
E-mail: brlee@skku.edu

S. Lee, H. Song, J. W. Yoon, Prof. H. Choi
Department of Chemistry, Research Institute for Convergence of Basic Sciences, and Research Institute for Natural Science, Hanyang University
Seoul 04763, Republic of Korea
E-mail: hschoi202@hanyang.ac.kr

S. Lee, Prof. S. H. Park,
Department of Physics, Pukyong National University
Busan 48513, Republic of Korea
E-mail: spark@pknu.ac.kr

S. Lee, Prof. S. H. Park
CECS Research Institute, Core Research Institute, Busan 48513, Korea

Dr. X. Shen, Prof. Henry J. Snaith
Clarendon Laboratory, Department of Physics, University of Oxford, Oxford, UK

H. Choi, Y. Choi, Prof. H. Y. Kim
Department of Materials Science and Engineering, Chungnam National University, Daeduk Science Town, Daejeon 34134, Republic of Korea

Dr. J. Yang
Institute for Advanced Materials and Manufacturing, Department of Materials Science and Engineering, University of Tennessee, Knoxville, TN 37996, United States

Dr. J. Lee
School of Earth and Environmental Sciences, Seoul National University, Seoul 08826, Republic of Korea

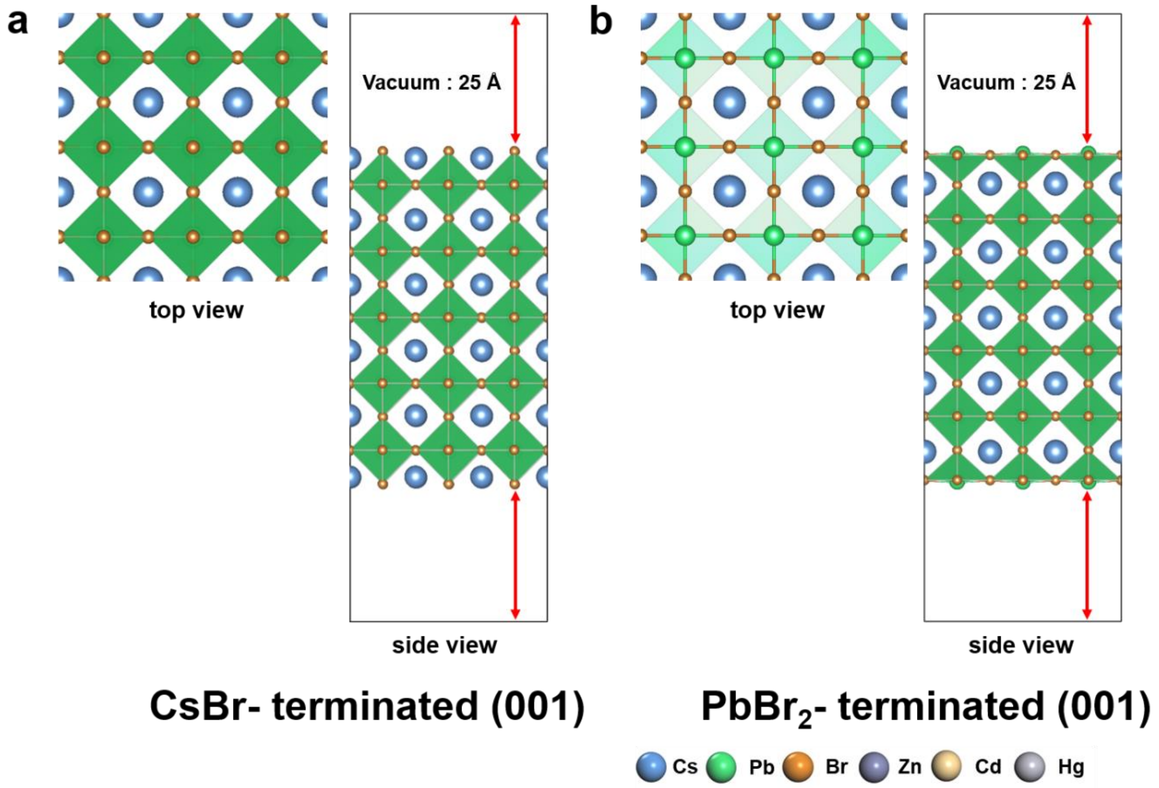

Figure S1. The DFT-constructed slabs a) CsBr-terminated (001), b) $PbBr_2$-terminated (001).

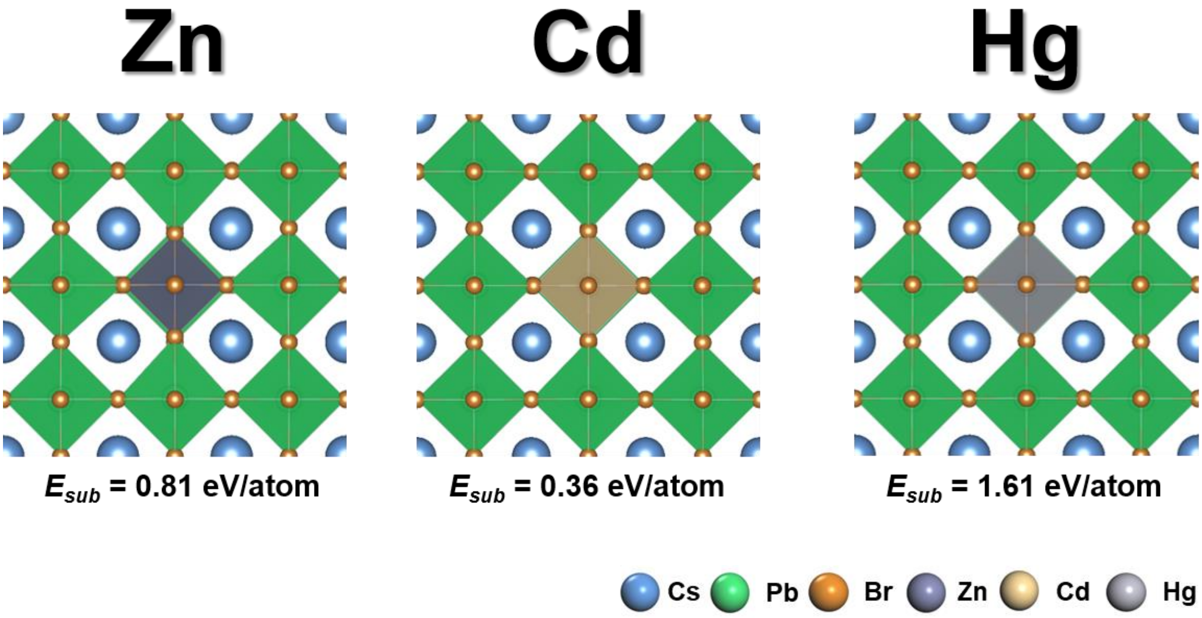

Figure S2. The DFT-estimated substitutional energies (E_{sub}) over the CsBr-terminated (001).

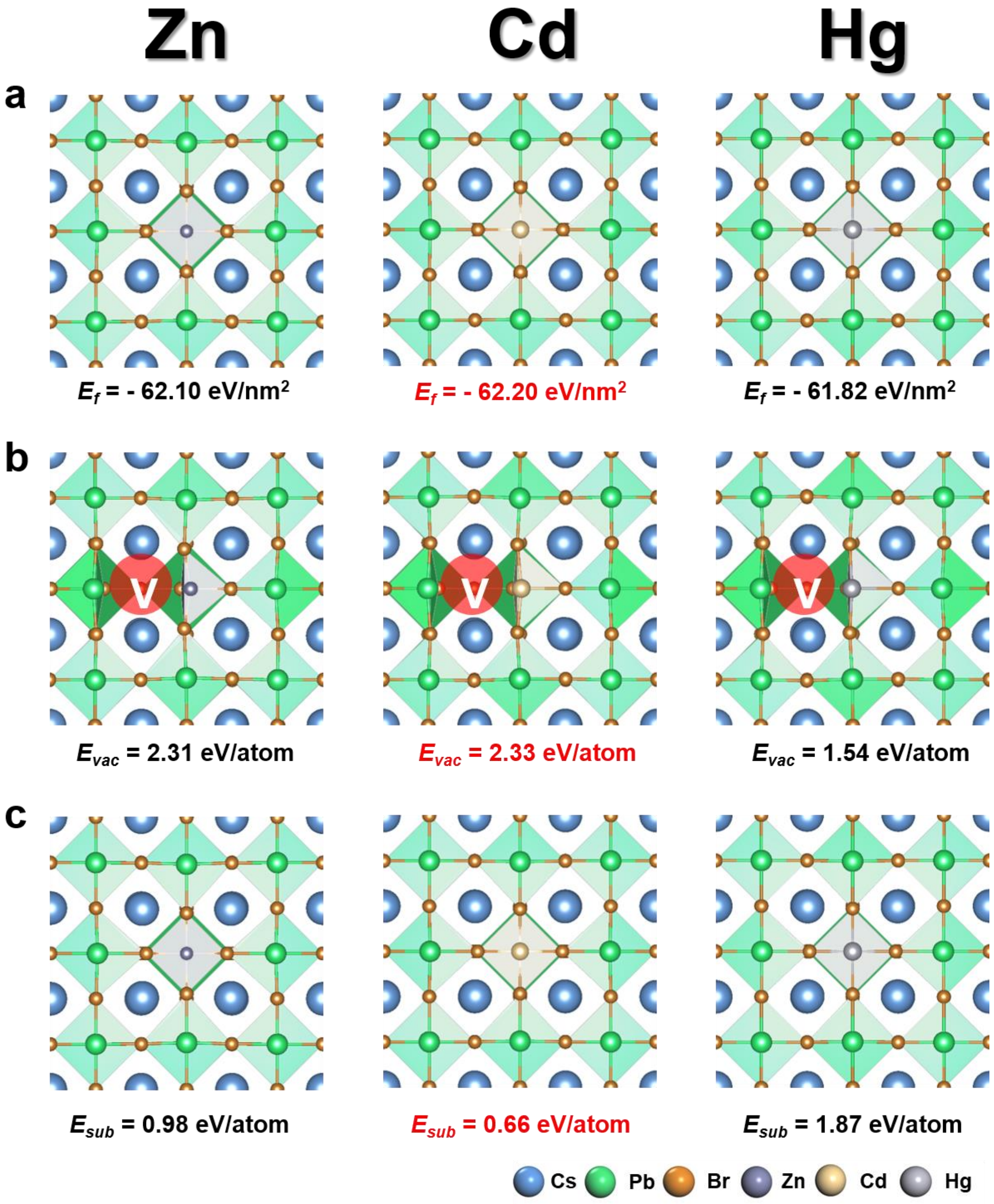

Figure S3. The DFT-calculated energies corresponding to structural stability over the $PbBr_2$-terminated (001). a) Surface formation energies (E_f) b) Defect formation energies (E_{vac}). The highlighted red circles denote the Br vacancy sites on the surface c) Substitutional energies for metal-doped surfaces (E_{sub}).

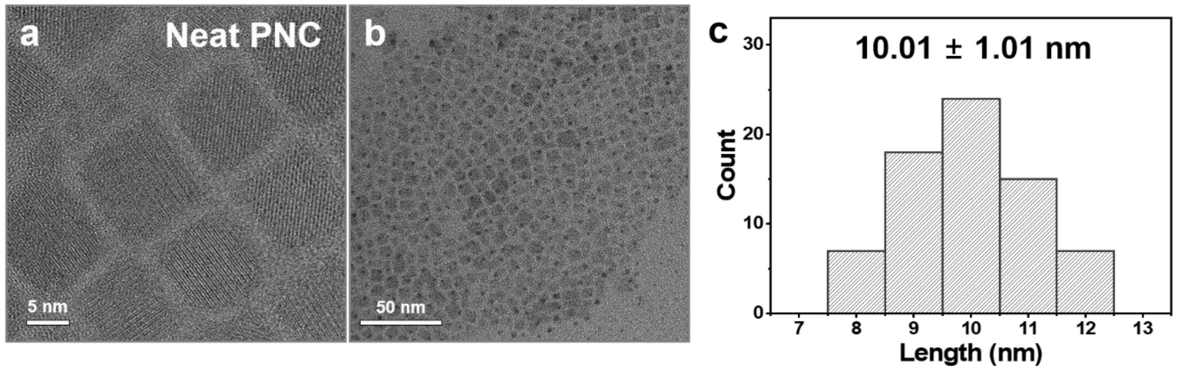

Figure S4. TEM images at a) high magnification and b) low magnification, c) Histogram of size with neat PNCs.

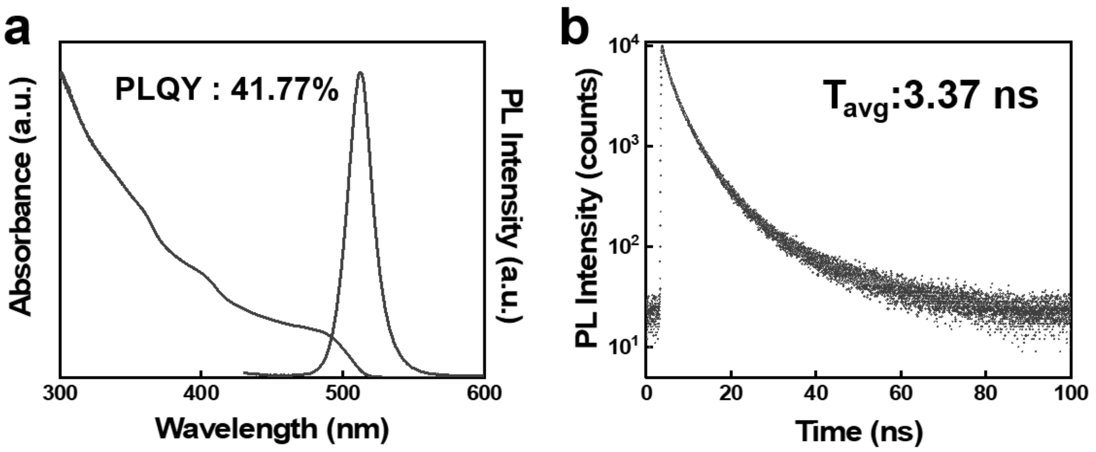

Figure S5. a) Absorption and PL spectrum, b) Time-resolved PL decay curves of neat PNCs.

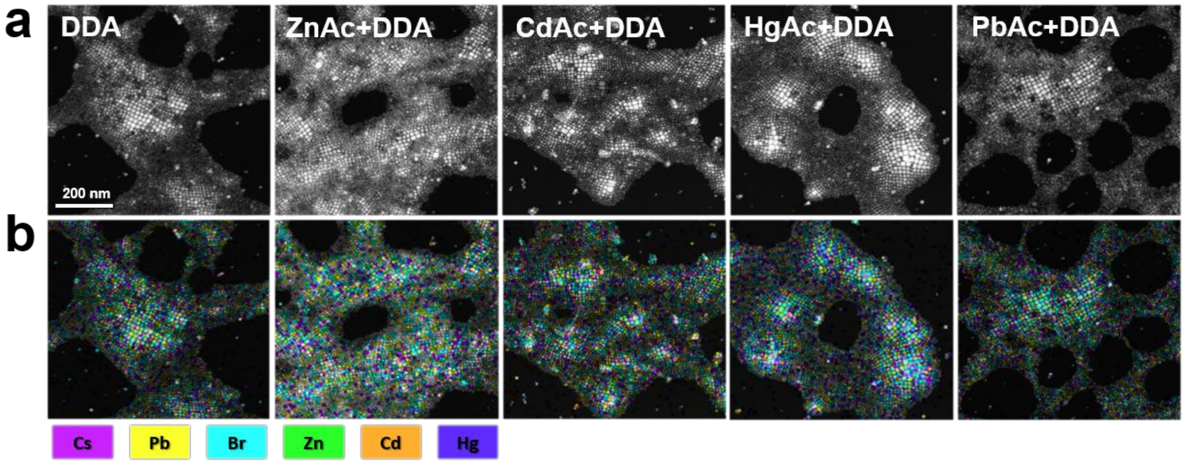

Figure S6. a) Low magnification TEM image of DDA- and Metal acetate+DDA PNCs. b) EDS mapping images of Cs, Pb, Zn, Cd, Hg and Br atoms.

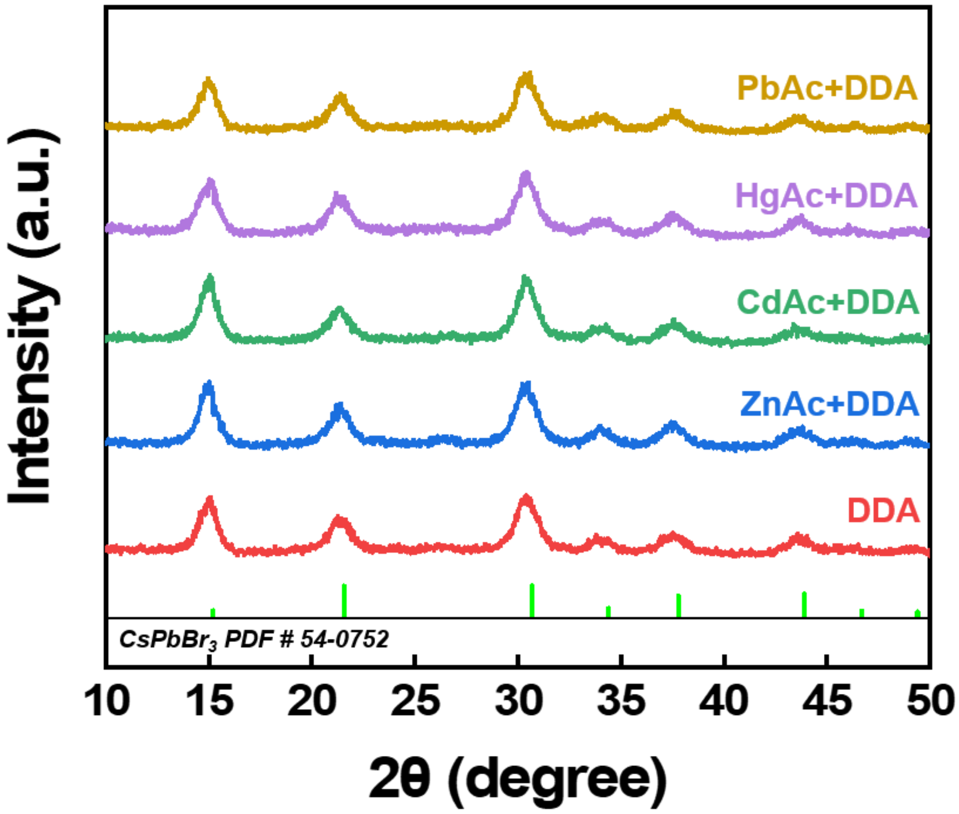

Figure S7. Powder X-ray diffraction pattern of PNCs.

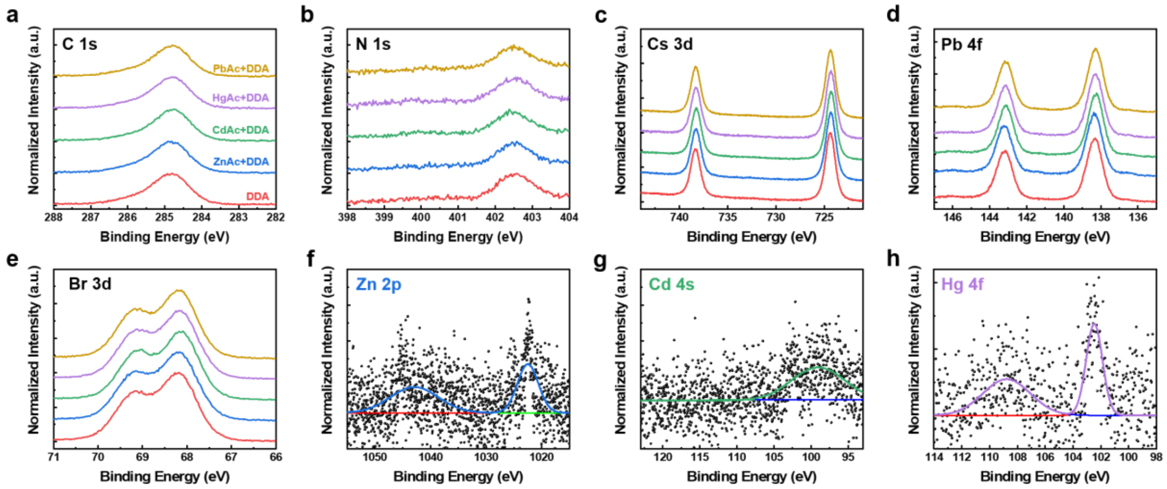

Figure S8. XPS spectra of each PNCs. a) C 1s, b) N 1s, c) Cs 3d, d) Pb 4f, e) Br 3d, f) Zn 2p, g) Cd 4s, h) Hg 4f.

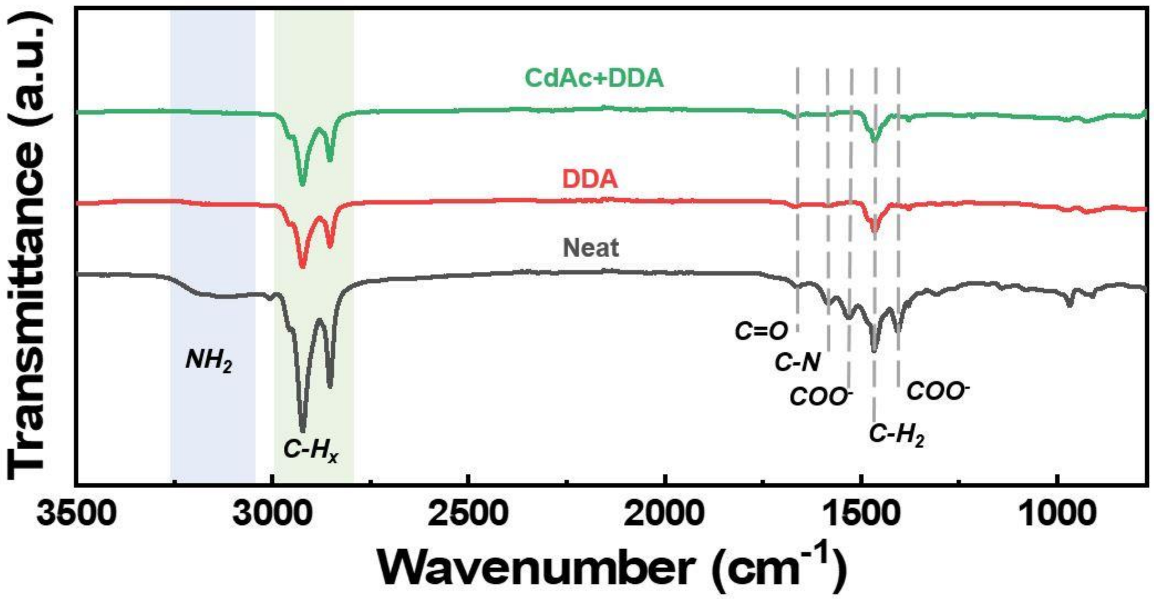

Figure S9. FT-IR spectra of each PNCs. Frequencies for expected functional groups in each ligand are indicated.

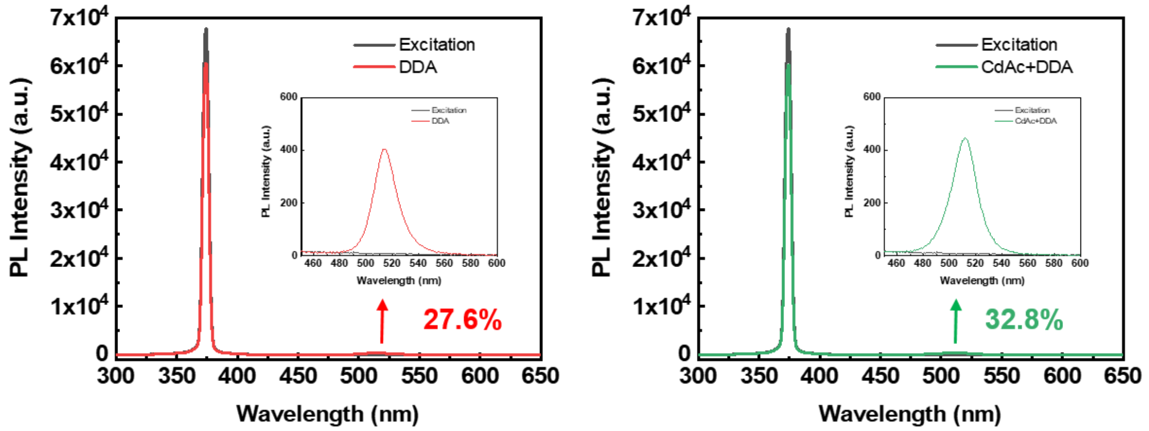

Figure S10. PLQY spectra of DDA PNC and CdAc+DDA PNC films deposited on the glass substrate.

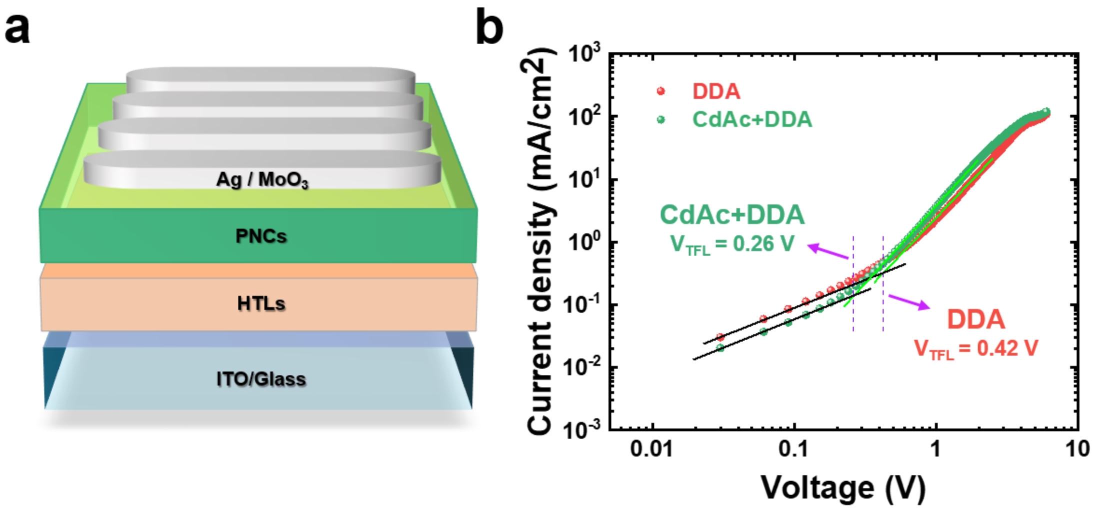

Figure S11. a) Hole-only device architecture, b) *J-V* characteristics of hole-only devices.

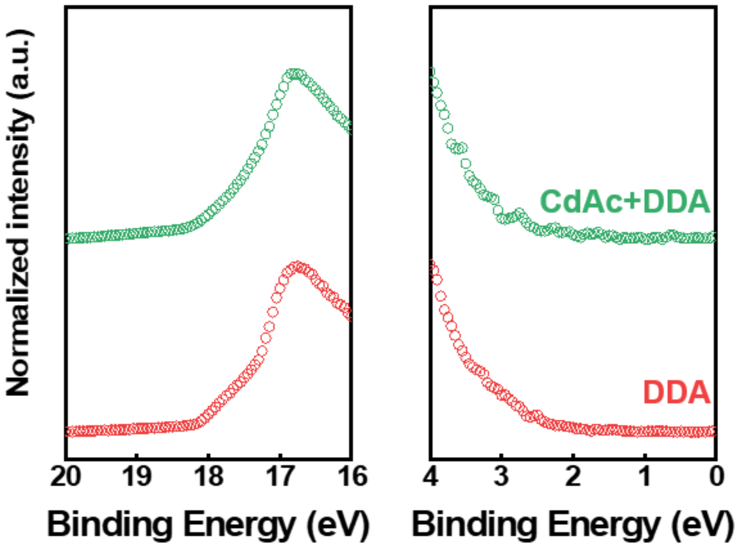

Figure S12. UPS spectra of each PNC films.

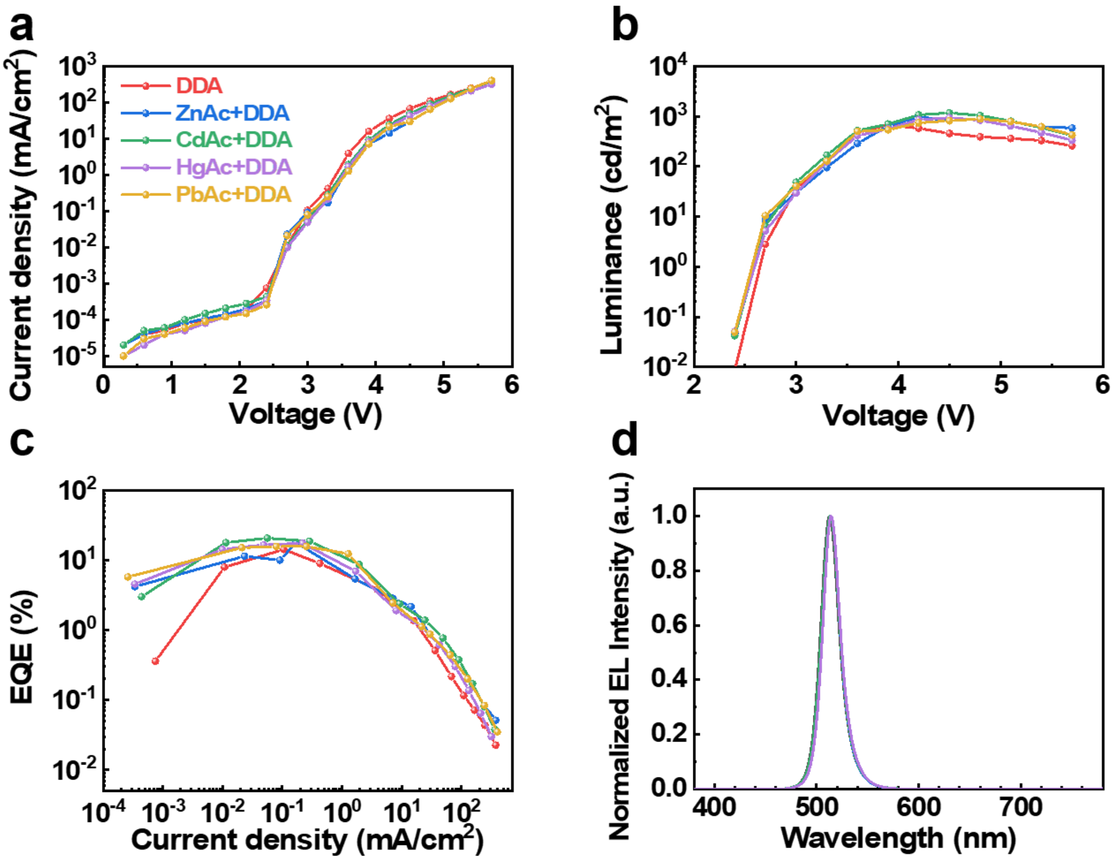

Figure S13. a) Current density–voltage, b) Luminance–voltage, c) Current density–EQE. d) Normalized electroluminescence spectrum of each PeLED device.

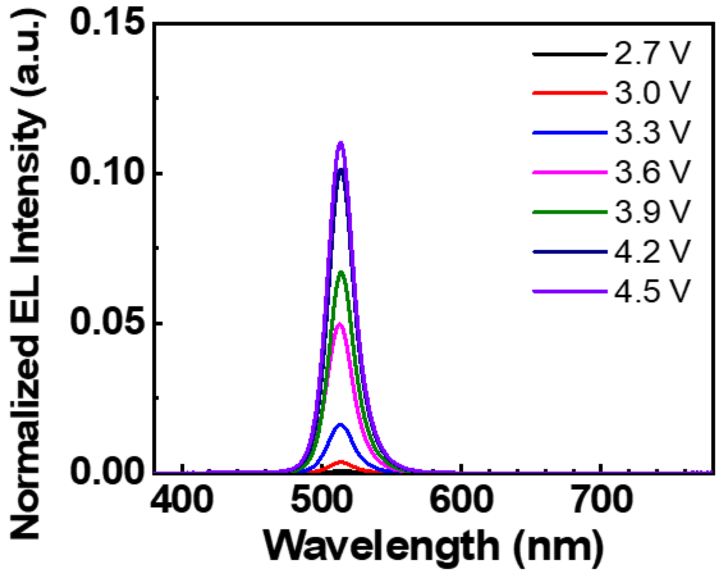

Figure S14. Electroluminesence spectra of CdAc+DDA PeLED under different biases.

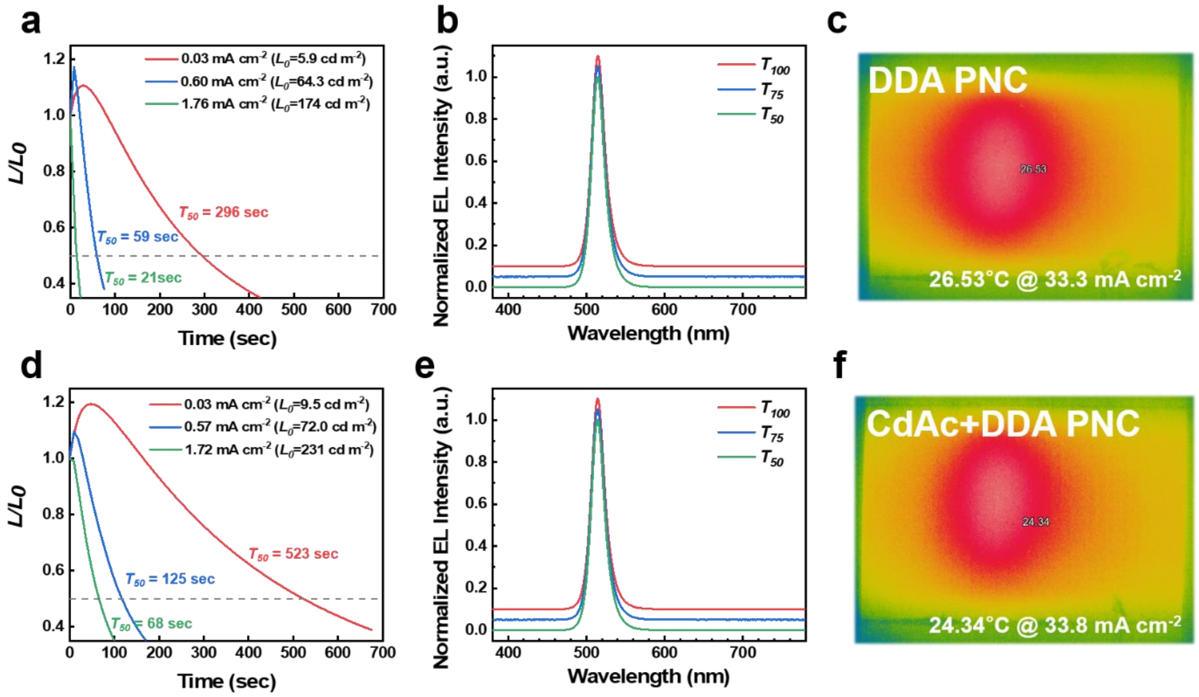

Figure S15. Device stability test from lifetime measurements (T_{50}) of a) DDA and d) CdAd+DDA PNC device, b,e) Electroluminesence spectra of each step, c,f) Temperature image of each PeLEDs operated image using a near-infrared camera.

Table S1 XPS analysis of each PNCs.

Sample configuration	Actual atomic ratio in PNCs (Metal : Pb; %)
ZnAc+DDA PNC	1.10
CdAc+DDA PNC	1.33
HgAc+DDA PNC	1.09

Table S2 Time-resolved PL decay profiles of perovskite NC solutions.

Sample configuration	τ_1 (ns)	A_1 (%)	τ_2 (ns)	A_2 (%)	τ_3 (ns)	A_3 (%)	τ_{avg} (ns)
DDA PNC	3.50	27.99	8.82	55.77	19.32	16.24	9.04

ZnAc+DDA PNC	4.49	28.35	10.58	55.80	22.09	15.85	10.68
CdAc+DDA PNC	4.72	31.05	11.30	57.04	24.28	11.91	10.80
HgAc+DDA PNC	4.63	31.88	10.81	52.21	22.08	15.91	10.63
PbAc+DDA PNC	3.80	34.79	10.41	19.57	21.24	19.57	10.23

τ_1, τ_2, and τ_3: Lifetimes A_1, A_2 and A_3: Respective fractional contributions

τ_{avg}: Average lifetime (τ_{avg}) which is calculated using $\tau_{avg} = \sum_{i=1}^{3} A_i \cdot \tau_i$

Table S3. Summarized device performances of PeLEDs.[a]

Sample configuration	L $_{max}$ [cd/m^2] @ bias	CE $_{max}$ [cd/A] @ bias	EQE $_{max}$ [%] @ bias	Turn-on Voltage [V] @ 0.1cd/m^2	Wavelength [nm]
DDA PNC	684@3.9	49.08@3.0	14.32@3.0	2.7	514
ZnAc+DDA PNC	985@4.2	56.08@3.3	17.83@3.3	2.7	514
CdAc+DDA PNC	1175@4.5	65.48@3.0	20.79@3.0	2.7	514
HgAc+DDA PNC	940@4.5	55.88@3.3	17.50@3.3	2.7	514
PbAc+DDA PNC	887@4.8	51.00@3.0	15.76@3.0	2.7	514

[a]Full device : ITO / PEDOT:PSS / TFB / PTAA / PNC / TPBi / LiF / Al

Table S4. Summary of Green emissive $CsPbBr_3$ based perovskite NCs LED performance parameters in the literature.

Perovskite	EL peak [nm]	EQE [%]	CE$_{max}$ [cd/A]	L$_{max}$ [cd/m^2]	Reference
Engineering with TOAB, DDAB, OTAc ligand, FA doped $CsPbBr_3$	515	11.6	45.4	55800	Adv. Mater. **2018** 30, 1800764
Organic(OTAc,DDAB), Inorganic (Zn) passivation	518	16.48	66.7	76940	Adv. Mater. **2018** 30, 1805409
NABr modified	512	17.4	54.6	8353	Adv. Opt. Mater. **2019** 7, 190074

NiO_x decorated NC	512	16.8	32.4	2114	Nanoscale<br>**2020** 12, 8711-8719
BDABr treated NC	509	8.56	25.5	14021	Nano Energy<br>**2020** 70, 104467
Bipolar-shell resurfacing	~510	22	-	1200	Nat. Nanotechnol.<br>**2020** 15, 668-674
Fluoride treated	511	19.34	-	2030	Nat. Photonics<br>**2021** 15, 379-385
Ag-TOP treatment	511	9.43	27.35	3820	Chemical Engineering Journal<br>**2021** 414, 128866
Betain passivation	518	10.8	-	1075	Chemical Engineering Journal<br>**2023** 453, 139909
Ultrathin $CsPbBr_3$:NiO_x	514	26.7		13100	ACS Energy Lett.<br>**2023**, 8, 927−934
Zn FA doped $CsPbBr_3$	518	20.18	-	23800	ACS Energy Lett.<br>**2023**, 8, 1445-1454
CdAc+DDA treated	514	20.79	65.48	1175	This work
